# Whole-Genome Sequences of Novel Porcine Circovirus Type 2 Viruses Detected in Swine from Mexico and the United States

**DOI:** 10.1128/genomeA.01315-15

**Published:** 2015-12-17

**Authors:** Karen M. Harmon, Phillip C. Gauger, Jianqiang Zhang, Pablo E. Piñeyro, Derek D. Dunn, Amy J. Chriswell

**Affiliations:** Department of Veterinary Diagnostic and Production Animal Medicine, College of Veterinary Medicine, Iowa State University, Ames, Iowa, USA

## Abstract

A unique porcine circovirus type 2 capsid protein (ORF2) sequence was detected in swine samples submitted to the Iowa State University Veterinary Diagnostic Laboratory. The complete genome sequences of four viruses, one from Mexico and three from the United States, were determined to further characterize this novel PCV2 genotype.

## GENOME ANNOUNCEMENT

Porcine circovirus type 2 (PCV2) is a small, nonenveloped, single-stranded DNA virus belonging to the family *Circoviridae* and genus *Circovirus* (1). Porcine circovirus type 2 is the cause of several syndromes in swine known en masse as porcine circovirus-associated disease (PCVAD) or porcine multisystemic wasting syndrome (2–4). Currently, four global PCV2 genotypes have been described in swine based on the ORF2 capsid protein sequence and designated PCV2a, PCV2b, PCV2c, and PCV2d (formerly mutant PCV2b) (5).

Over the course of approximately 1 year, four different cases submitted to the Iowa State University Veterinary Diagnostic Laboratory (ISU VDL) were tested for PCV2 by PCR and demonstrated atypical nucleotide and amino acid sequences of the ORF2 (Cap) protein. Specimen types were serum from a sow (Mexico), serum from placental umbilical cord (USA, state unknown), and two lungs (North Carolina and Indiana). By use of traditional Sanger sequencing, the full genome of PCV2 was determined in each submission. SeqManPro and Megalign (Clustal W) modules from DNAStar program were used for assembly of contigs and sequence comparisons.

The full-length genomic sequences of PCV2/MEX/41238/2014, PCV2/USA/34701/2015, PCV2/USA/43520/2015, and PCV2/USA/45358/2015 are 1,777 nucleotides (nt) in length, in contrast to 1,767 nt for typical PCV2, and have 99.35%–99.7% nt identity with each other. At the whole-genome level, the four sequences described here have similar genomic organization to previously described PCV2 isolates. BLAST searches using these four PCV2 whole-genome sequences resulted in numerous hits of 93% or lower nucleotide homology with other PCV2 whole-genome sequences.

The ORF1 (encoding a putative replication initiator protein) sequence from each of the four PCV2 is 945 nt, consistent with the size of the ORF1 of other PCV2 sequences. BLAST searches with these ORF1 sequences resulted in numerous hits with up to 99% nt identity to other PCV2 ORF1 sequences.

The four PCV2 ORF2 sequences, encoding a putative Cap protein, are 717 nt in contrast to 702 nt for reported PCV2a or PCV2b, or 705 nt for reported PCV2c or PCV2d. Compared to PCV2a or PCV2b, all four sequences reported here contain a 15-nt insertion close to the 3′ end of ORF2, resulting in the insertion of five amino acids, Pro–Leu–Pro–Tyr–Met or Pro–Leu–Ser–Tyr–Met. Compared to PCV2c or PCV2d, these unique sequences have 12 additional nt, encoding Pro–Leu–Pro(Ser)-Tyr. The closest ORF2 sequence available in GenBank (accession number JN382183) using the putative Cap sequences of each of these PCV2 demonstrated 96% coverage and only 88% nt identity.

The four unique sequences were compared to reference PCV2 genomes representing the four current phylogenetic clusters based on the global distribution of PCV2 genotypes (6). Sequence analysis of the whole genome and ORF2 demonstrated that these four sequences form their own separate genetic clade. The ORF2 sequences were closest to PCV2c and PCV2d (84% to 85.8% nt identity). At the amino acid (aa) level, they were closest to PCV2c (86.0% to 87.2% identity).

The association of these unique PCV2 sequences with clinical PCVAD remains unknown. Further analysis and porcine bioassays are required to determine the clinical significance of this novel PCV2 genotype.

### Nucleotide sequence accession numbers.

The complete genome sequences of PCV2/MEX/41238/2014, PCV2/USA/34701/2015, PCV2/USA/43520/2015, and PCV2/USA/45358/2015 have been deposited in GenBank under the accession numbers KT795287, KT795288, KT795289, and KT795290, respectively.

## References

[1] Tischer I, Gelderblom H, Vettermann W, Koch MA 1982 A very small porcine virus with circular single-stranded DNA. Nature 295:64–66. doi:10.1038/295064a0. 7057875

[2] Gillespie J, Opriessnig T, Meng XJ, Pelzer K, Buechner-Maxwell V 2009 Porcine circovirus type 2 and porcine circovirus-associated disease. J Vet Intern Med 23:1151–1163. doi:10.1111/j.1939-1676.2009.0389.x. 19780932 PMC7166794

[3] Harding J, Clark E 1997 Recognizing and diagnosing postweaning multisystemic wasting syndrome (PMWS). Swine Health Prod 5:201–203.

[4] Segalés J 2012 Porcine circovirus type 2 (PCV2) infections: clinical signs, pathology and laboratory diagnosis. Virus Res 164:10–19. doi:10.1016/j.virusres.2011.10.007. 22056845

[5] Franzo G, Cortey M, Olvera A, Novosel D, de Castro AMMG, Biagini P, Segalés J, Drigo M 2015 Revisiting the taxonomical classification of porcine circovirus type 2 (PCV2): still a real challenge. Virol J 12:131. doi:10.1186/s12985-015-0361-x. 26311322 PMC4551364

[6] Xiao C, Halbur PG, Opriessnig T 2015 Global molecular genetic analysis of porcine circovirus type 2 (PCV2) sequences confirms the presence of four main PCV2 genotypes and reveals a rapid increase of PCV2d. J Gen Virol 96:1830–1841. doi:10.1099/vir.0.000100. 25711965

